# Interventions Aiming to Promote Active Commuting in Children and Adolescents: An Evaluation From a Sex/Gender Perspective

**DOI:** 10.3389/fspor.2020.590857

**Published:** 2020-11-26

**Authors:** Isabel Marzi, Sandra Emmerling, Yolanda Demetriou, Jens Bucksch, Carolin Schulze, Catherina Brindley, Anne Kerstin Reimers

**Affiliations:** ^1^Department of Sport Science and Sport, Friedrich-Alexander-University Erlangen-Nuremberg, Erlangen, Germany; ^2^Institute of Human Movement Science and Health, Chemnitz University of Technology, Chemnitz, Germany; ^3^Department of Sport and Health Sciences, Technical University of Munich, Munich, Germany; ^4^Department of Natural and Sociological Sciences, Heidelberg University of Education, Heidelberg, Germany

**Keywords:** active commuting, girls, boys, equity, randomized controlled trails, sex/gender checklist

## Abstract

Active commuting (AC) provides numerous health benefits and is one way to improve physical activity in children and adolescents. Boys are more likely to use active transport modes than girls. Girls and boys benefit differently from interventions that promote AC. The aim of this systematic review is to evaluate the effects of interventions on girls and boys and to appraise the extent to which previous studies have taken sex/gender into account. Eleven electronic databases were searched to identify all relevant randomized and non-randomized controlled trials based on *a priori* defined eligibility criteria. Two independent reviewers screened the literature for eligibility and assessed risk of bias. Semiquantitative analyses were conducted to evaluate the effects of intervention effects by taking sex/gender aspects into account. To evaluate sex/gender considerations in interventional studies, a recently developed sex/gender checklist was applied. Twelve studies were included that examined intervention effects on AC in girls and boys. Three intervention studies showed significant effects in increasing AC, with one study favoring girls, one favoring boys, and another focusing on a single sex/gender (only girls). According to the checklist, the overall sex/gender rating highlighted a lack of information in sex/gender consideration. Studies with and without significant effects indicated no differences in the sex/gender checklist. The results indicate that sex/gender is not considered adequately in primary interventional research on AC. To evaluate the effectiveness of intervention in boys and girls, detailed analyses of sex/gender are required, and better reporting about sex/gender-specific intervention content is necessary. In future health research to promote AC, sex/gender should be systematically taken into account.

## Introduction

Active commuting (AC) on a regular basis provides several health benefits for children and adolescents. AC increases mental well-being (Hamer et al., 2009); helps prevent cardiovascular disease, obesity, arthritis, depression, diabetes, and anxiety disorders (Mason, 2000; Penedo and Dahn, 2005); and can improve children's academic performance (Dwyer et al., 2001) and body composition (Garrard, 2009). Furthermore, a variety of benefits of AC can be found more generally such as a decline in carbon emissions, less traffic noise, greater social interaction (Bauman et al., 2008), and a reduction in injury rates (Garrard, 2009).

Forms of AC during childhood and adolescence include walking, cycling, skateboarding, skating, or scooter riding to neighborhood destinations such as school, shops, sports facilities, and/or friends' homes (Timperio et al., 2004; Carver et al., 2010; Nelson and Woods, 2010). It appears that AC is associated with greater overall physical activity (PA) in boys and girls (Oreskovic et al., 2014; Ogilvie et al., 2016; Dinu et al., 2019). Therefore, promoting AC could be a simple way to foster PA and reduce sedentary behavior in children and adolescents (McCormack et al., 2011; Mendoza et al., 2011). Empirical evidence indicates that inactive commuting behaviors established in childhood and adolescence continue into adulthood and have lasting effects on one's health and well-being (Telama et al., 2014; Yang et al., 2014). Therefore, children and adolescents are an important target group for interventions promoting AC.

Despite the known health benefits, AC has declined over the last decades (Johansson et al., 2012). The number of children and adolescents who actively commute to school on a regular basis decreased worldwide over the last 50 years. In the US, declines ranged from 41% in 1969 to 13% in 2009 (McDonald et al., 2011). In 5–9-years-olds from Australia, active commuting to school (ACS) declined from 58% in 1971 to 25% in 2003 (van der Ploeg et al., 2008). In Canada, there was a decline in ACS from 53% to 42% between 1986 and 2006 (Buliung et al., 2009). Similarly, AC declines in Europe. In urban areas in Switzerland, ACS decreased significantly from 78% in 1994 to 72% in 2005 (Grize et al., 2010). In high-income countries, there is a general trend toward an increasing use of motorized transport (McDonald, 2007; Grize et al., 2010).

Additionally, it has been shown that in most countries, AC and ACS are less prevalent in girls than in boys (McDonald, 2007; Chillon et al., 2010; Oreskovic et al., 2014). Boys are more likely to cycle to school (Oreskovic et al., 2014; Carver et al., 2015; Kallio et al., 2016), while girls are more likely to walk (McDonald, 2007; Chillon et al., 2010; Panter et al., 2010). Furthermore, boys accumulate more steps than girls while walking to school (Craig et al., 2010; McCormack et al., 2011). Differences in AC levels between girls and boys can be explained by socialization theory (Hurrelmann and Bauer, 2015) and by social constructive theories (for example, “doing gender”) (West and Zimmermann, 1987). These gender theories postulate that differences occur due to socially determined gender roles (West and Zimmermann, 1987). Furthermore, socialization processes constitute gender-typed patterns of behavior (Ristvedt, 2014; Kilvington and Wood, 2016). Social and cultural norms, biological mechanisms, and genetic dispositions can contribute to the sex/gender-specific differences in the level of PA in children and adolescents (Telford et al., 2016). The term “gender” refers to the “socially prescribed and experienced dimensions of ‘femaleness’ or ‘maleness’ in a society” (Johnson et al., 2009), while the term “sex” refers to biological and physiological processes (Johnson et al., 2009). Both influences of sex hormones on PA (Bowen et al., 2011) and environment-induced gender differences in PA participation (Thomas and Thomas, 1988; Schmalz and Kerstetter, 2006) have been reported.

In terms of ACS, parents generally seem to be more protective over their daughters than their sons (Brown et al., 2008). In particular, mothers tend to consider girls as more vulnerable than boys and therefore have higher safety concerns (Brown et al., 2008). Consequently, girls are not allowed the same degree of independent mobility as boys (O'Brien et al., 2000; Brown et al., 2008), while boys enjoy a comparatively higher level of independence (Brown et al., 2008). Furthermore, stranger danger and road safety concerns limit AC in both girls and boys (Garrard, 2009). Differences in the mode of travel between males and females persist until adulthood, with men being more likely to cycle to work and women commuting by car (Simons et al., 2017). It has been shown that women perceive their environment as more insecure than men (Akar et al., 2013). This may well-contribute to the fact that women cycle less frequently than men and tend to use cars or public transport. A previous analysis indicates that 37% of women do not use their bike if no bike path is available (Akar et al., 2013).

Research in AC, especially in ACS, has expanded in past years but has thus far not considered sex/gender differences systematically (Chillón et al., 2011; Larouche et al., 2018; Villa-Gonzalez et al., 2018; Schönbach et al., 2020). Current systematic reviews revealed that the majority of included intervention studies reported modest effect sizes on ACS, were of poor quality, and did not account for aspects of sex/gender (Larouche et al., 2018; Villa-Gonzalez et al., 2018). A systematic review by Carlin et al. (2016) did, however, aim to analyze sex/gender differences in walking interventions but did not apply any criteria to assess these differences. As a result, there was a lack of evidence regarding the effectiveness of AC interventions in relation to sex/gender.

The aim of this current systematic review is to evaluate the effects of interventions that promote AC in children and adolescents by systematically taking aspects of sex/gender into account. On the basis of a currently developed sex/gender checklist, this paper fills a knowledge gap and summarizes the effectiveness of interventions promoting AC in terms of similarities and differences in boys and girls. This systematic review aims to present a current knowledge base to inform and help policymakers and stakeholders to develop interventions to promote AC.

## Methods

The current study is part of the collaborative genEffects project that evaluates the effects of interventions on girls' and boys' PA and sedentary behavior and is reported according to the Preferred Reporting Items for Systematic reviews and Meta-Analyses (PRISMA) guidelines (Liberati et al., 2009). Due to the high number and heterogeneity of studies, the included intervention studies in the genEffects systematic review were split into five categories according to the following PA outcomes: (1) PA at school, (2) leisure time PA, (3) AC, (4) sedentary behavior, and (5) overall PA. The current systematic review focuses on interventions that promote AC, and therefore, only primary studies with AC as one outcome were included. The systematic review protocol of the genEffects project has been published previously (Demetriou et al., 2019) and is registered (ref CRD42018109528). There were no protocol amendments, except for the GRADE framework, which was not used due to the semiquantitative analyses of data. Additionally, we were unable to conduct a meta-analysis as planned (Demetriou et al., 2019) due to the heterogeneity of primary studies concerning methodologies and outcome measurements.

### Search Strategy and Eligibility Criteria

For the genEffects systematic review, a comprehensive literature search was conducted using 11 electronic databases [Cochrane Central Register of Trials (CENTRAL); U.S. National Library of Medicine (clinicalTrials.gov); Ovid Embase; Epistemonikos; EBSCO Eric; WHO International Clinical Trails Registry Platform (ICTRP); Ovid Medline; ProQuest Dissertations & These Global; EBSCO PsycINFO; EBSCO SPORTDiscus; Clarivate Web of Science] in August 2018. The search strategy of the genEffects project was based on Cochrane standards. The included primary studies had to fulfill the following criteria: Participants were healthy children and adolescents within the average age range of 3–19 years; the aim of the intervention had to be the promotion of PA and/or the reduction of sedentary behavior; the study was a (cluster-/randomized) controlled trial with a comparison group without components promoting PA and/or reducing sedentary behavior; the study was published as an English language peer-reviewed journal article after the year 2000; and one of the outcomes had to be AC assessed by any type of measure (subjective/objective) with any form of descriptive or inferential statistic. Furthermore, outcomes of AC had to be reported (I) for sex/gender disaggregated at baseline and/or follow-up, and/or it had to be explained (II) how they dealt with sex/gender during the outcome analysis (i.e., sex/gender adjusted analysis), and/or it had to be reported (III) that there were no differences in the outcome when looking at sex/gender. Additional articles were sought by reviewing reference lists of included full-text articles and systematic reviews of AC/ACS interventions.

### Study Selection and Data Extraction

The study selection for the genEffects systematic review was performed by two independent reviewers using Covidence systematic review software (Veritas Health Innovation, Melbourne, Australia; available at www.covidence.org). After de-duplication, titles and abstracts were screened, and articles of potential or indeterminate relevance were retrieved for full-text screening against eligibility criteria. All conflicts were resolved by a third senior researcher.

Data were extracted from each primary study and covered information about general study characteristics (country, design, name of intervention program), sample size for intervention and control groups stratified by sex/gender and dropout rates, details about intervention content, as well as intervention approaches and settings. Additionally, extraction forms contained information concerning interventions' main outcomes, measurement points and instruments, and statistical approaches, including confounders. All these aspects were relevant to the analysis of the effectiveness of these interventions. For additional information, study protocols and Supplementary Materials were used and, in case of missing information, the author(s) of the articles were contacted (maximum two contact attempts).

### Risk of Bias

Risk of bias was carried out independently by two reviewers using the Cochrane Collaboration's risk of bias tool for randomized trials (Higgins et al., 2011). Using the seven domains of the tool, primary studies were assessed for selection, performance, attrition, detection, reporting, and “other” bias. For “other” bias, we assessed baseline differences between intervention and control arms as well as seasonal differences in measurement points. Each domain was judged as “low,” “high,” or “unclear” risk of bias, with the last category indicating either lack of information or uncertainty about the potential for bias. Discrepancies were resolved through discussion or adjudication by a third reviewer. Review Manager 5 (The Nordic Cochrane Centre, 2014) tool was used to assess the overall risk of bias.

### Sex/Gender Assessment

To assess the degree to which sex/gender was considered in the included intervention studies, a recently developed sex/gender checklist was used (Demetriou et al., 2019). This sex/gender checklist was specially developed to rate the degree to which sex/gender aspects are considered in intervention studies. The checklist consists of 10 items analyzing sex/gender aspects in the five categories of *background and concepts, study design, intervention planning and delivery, presentation of findings*, and *interpretation of findings* (Supplementary Material 1). The items were rated using four grades: “not relevant,” “basic,” “detailed,” or “no information provided.” The rating “not relevant” was exclusively applied to studies that recruited only boys or only girls for categories that were considered less applicable to single sex/gender studies (e.g., provision of sex/gender-disaggregated data for participant flow). The additional grade “poor” was used for the item *definition and use of sex/gender terminology* if sex and gender terminologies are used interchangeably.

### Synthesis of Results

For semiquantitative analyses in which we analyzed intervention effects regarding the results of the sex/gender checklist, outcomes were split in the following three possible groups: (1) outcomes with similar significant intervention effects for boys and girls, (2) outcomes with the same or no significant intervention effects for boys and girls, and (3) outcomes with different intervention effects for boys and girls. Single sex/gender studies were evaluated separately. Sex/gender considerations were specified by calculating the sum of ratings for “detailed,” “basic,” “no information provided,” “poor,” and “not relevant” for every item on the checklist.

## Results

### Flowchart

**Figure 1:**
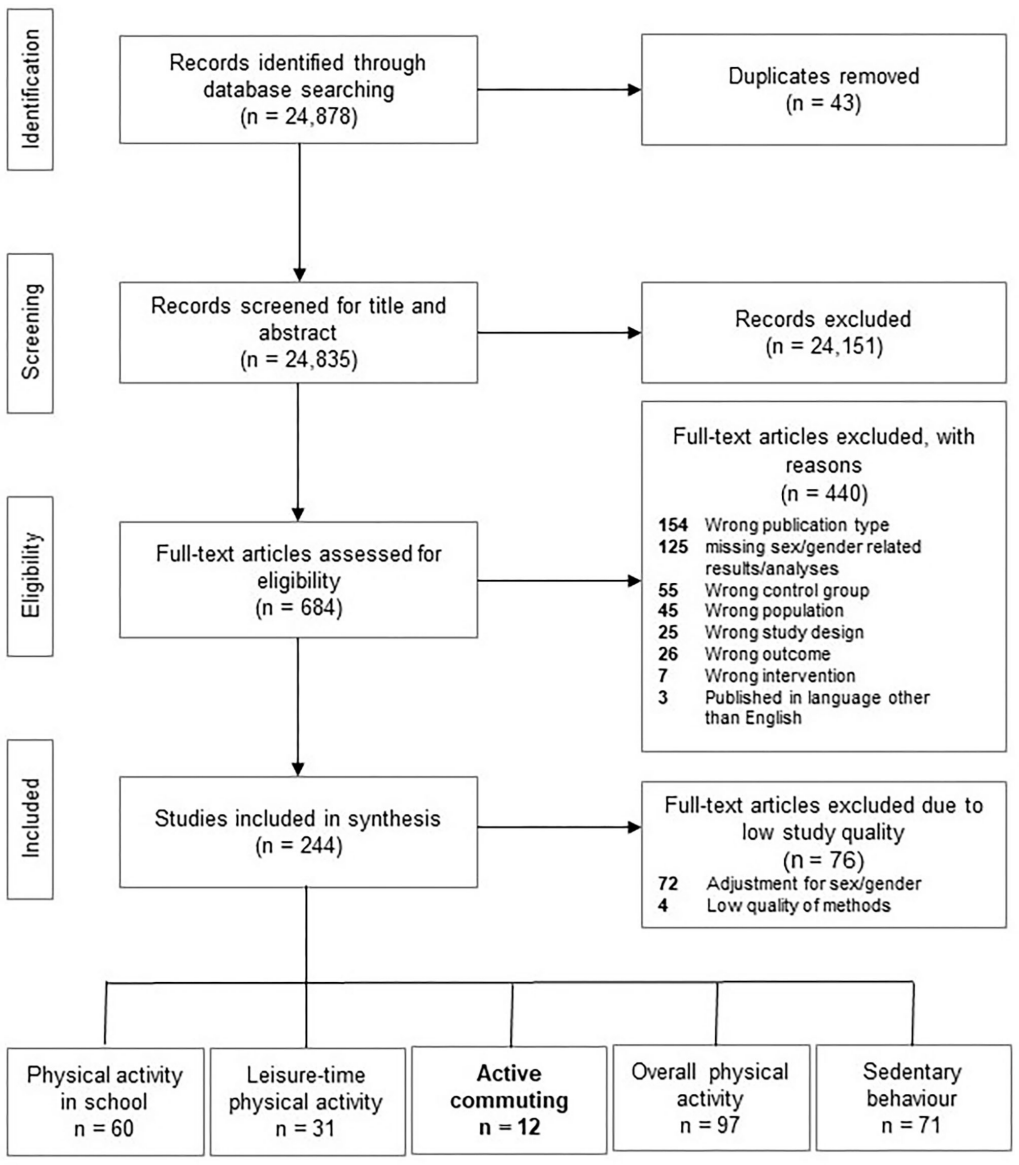
Flowchart.

In the genEffects project, out of 24,878 potentially relevant references identified by the electronic database searches, 244 studies were finally included (Figure 1). Out of these 244 studies, 12 intervention studies met the inclusion criteria and analyzed interventions on AC for sex/gender.

### Characteristics of the Included Studies

The characteristics of the included studies are presented in Table 1. A Supplemental Material shows further details of the included studies including study sample, intervention description, outcome measures, and results (see Supplementary Material 2). Most intervention studies were conducted in Europe, two in America (North and South) and in Australia/New Zealand, and one in Asia (China). Publication years ranged from 2007 to 2017. Eight of the included studies were cluster randomized controlled trials, while four were controlled trials. The sample size of the included interventions ranged from 97 (Duncan et al., 2011) to 2,434 (Haerens et al., 2007a) participants. Three studies focused on children (from 6 to 12 years) and nine studies on adolescents (from 13 to 19 years). All were applied in elementary or secondary schools. The duration of interventions ranged from 1 week (Bungum et al., 2014) to 20 months (van Nassau et al., 2014). In all intervention studies, the control group continued the usual practice and did not participate in any form of intervention. Only two studies treated AC/ACS as a primary outcome, whereas the other 10 studies focused on overall PA with AC/ACS as a secondary outcome. The majority of the studies examined ACS, two AC in general, and two AC in leisure time. In 11 studies, the assessement of AC occurred subjectively by questionnaires or diaries. AC was assessed objectively by observation and counting active commuters in one study only. Two studies comprised a single sex/gender study sample (one girls only, the other boys only), and five studies reported the results disaggregated for girls and boys. Four studies examined whether a significant interaction existed between allocation to intervention or control group, sex/gender, and time. One study statistically examined whether the effectiveness of the intervention differed between boys and girls (Cui et al., 2012). However, no differences were found, and therefore, the study did not report sex/gender-related results any further.

**Table 1:** Characteristics of included studies.

**Characteristics**	***n* (%)**	**Study source**
**Geographic Origin**		
Europe	7 (58)	Haerens et al., 2007a,b; Singh et al., 2009; Vasickova et al., 2013; Dubuy et al., 2014; van Nassau et al., 2014; Villa-Gonzalez et al., 2017
North/South America	2 (17)	Bungum et al., 2014; Filho et al., 2016
Australia/New Zealand	2 (17)	Duncan et al., 2011; Dewar et al., 2014
Asia	1 (8)	Cui et al., 2012
**Publication Year**		
2010–2017	9 (75)	Duncan et al., 2011; Cui et al., 2012; Vasickova et al., 2013; Bungum et al., 2014; Dewar et al., 2014; Dubuy et al., 2014; van Nassau et al., 2014; Filho et al., 2016; Villa-Gonzalez et al., 2017
2000–2009	3 (25)	Haerens et al., 2007a,b; Singh et al., 2009
**Study Design**		
Controlled trial	4 (33)	Bungum et al., 2014; Dubuy et al., 2014; van Nassau et al., 2014; Villa-Gonzalez et al., 2017
Cluster randomized controlled trial	8 (67)	Haerens et al., 2007a,b; Singh et al., 2009; Duncan et al., 2011; Cui et al., 2012; Vasickova et al., 2013; Dewar et al., 2014; Filho et al., 2016
**Sample Size**		
<500	6 (50)	Haerens et al., 2007a; Singh et al., 2009; Cui et al., 2012; Bungum et al., 2014; van Nassau et al., 2014; Filho et al., 2016
>500	6 (50)	Haerens et al., 2007b; Duncan et al., 2011; Vasickova et al., 2013; Dewar et al., 2014; Dubuy et al., 2014; Villa-Gonzalez et al., 2017
**Duration of Intervention**		
Short term (<3 months)	5 (42)	Haerens et al., 2007b; Duncan et al., 2011; Cui et al., 2012; Vasickova et al., 2013; Bungum et al., 2014
Moderate term (4–12 months)	6 (50)	Haerens et al., 2007a; Singh et al., 2009; Dewar et al., 2014; Dubuy et al., 2014; Filho et al., 2016; Villa-Gonzalez et al., 2017
Long term (>12 months)	1 (8)	van Nassau et al., 2014
**Setting**		
School	12 (100)	Haerens et al., 2007a,b; Singh et al., 2009; Duncan et al., 2011; Cui et al., 2012; Vasickova et al., 2013; Bungum et al., 2014; Dewar et al., 2014; Dubuy et al., 2014; van Nassau et al., 2014; Filho et al., 2016; Villa-Gonzalez et al., 2017
**AC Outcome**		
ACS	8 (67)	Singh et al., 2009; Duncan et al., 2011; Cui et al., 2012; Bungum et al., 2014; Dubuy et al., 2014; van Nassau et al., 2014; Filho et al., 2016; Villa-Gonzalez et al., 2017
AC in leisure time	2 (17)	Haerens et al., 2007a,b
AC in general	2 (17)	Vasickova et al., 2013; Dewar et al., 2014
**AC Measurement**		
Objective	1 (8)	Bungum et al., 2014
Subjective	11 (92)	Haerens et al., 2007a,b; Singh et al., 2009; Duncan et al., 2011; Cui et al., 2012; Vasickova et al., 2013; Dewar et al., 2014; Dubuy et al., 2014; van Nassau et al., 2014; Filho et al., 2016; Villa-Gonzalez et al., 2017
**Analyses of Sex/Gender**		
Single sex/gender	2 (17)	Dewar et al., 2014; Dubuy et al., 2014
Sex/gender disaggregated	5 (42)	Singh et al., 2009; Vasickova et al., 2013; Bungum et al., 2014; van Nassau et al., 2014; Villa-Gonzalez et al., 2017
Interaction	4 (34)	Haerens et al., 2007a,b; Duncan et al., 2011; Filho et al., 2016
Tested–no statistical results	1 (8)	Cui et al., 2012

### Risk of Bias Assessment

The results of the risk of bias assessment are presented in Table 2. Eleven included studies were judged to be at a high risk of bias in at least one domain. Risk of bias in primary studies shows a heterogeneous picture. Low risk of bias across all domains was found in 37% and a high risk of bias in 36%, and 27% of studies showed an unclear risk of bias. Determining domain-specific rating, selective reporting was at low risk of bias in all studies. Blinding of outcome assessment had the largest number of high-risk studies (60%).

**Table 2:** Risk of bias assessment for included studies.

**Study**	**Random sequence generation (selection bias)**	**Allocation concealment (selection bias)**	**Blinding of participants and personnel (performance bias)**	**Blnding of outcome assessment (detection bias)**	**Incomplete outcome data (attrition bias)**	**Seletive reporting (reporting bias)**	**Other bias**
Bungum et al. (2014)	High	High	High	High	Unclear	Low	Low
Cui et al. (2012)	Low	High	Unclear	Unclear	Low	Low	Low
Dewar et al. (2014)	Unclear	Low	Unclear	Low	Low	Low	Low
Dubuy et al. (2014)	High	High	High	High	Low	Low	Unclear
Duncan et al. (2011)	Unclear	Unclear	Unclear	Low	High	Low	Unclear
Filho et al. (2016)	Unclear	Low	Unclear	High	Low	Low	Low
Haerens et al. (2007a)	Unclear	Unclear	High	Low	Low	Low	Unclear
Haerens et al. (2007b)	High	Unclear	High	High	Low	Low	Low
Singh et al. (2009)	Unclear	Unclear	Unclear	High	Low	Low	High
van Nassau et al. (2014)	High	High	Unclear	High	Low	Low	High
Vasickova et al. (2013)	Unclear	Unclear	High	High	High	Low	High
Villa-Gonzalez et al. (2017)	High	High	Unclear	High	High	Low	High

### Sex/Gender Checklist

**Figure 2:**
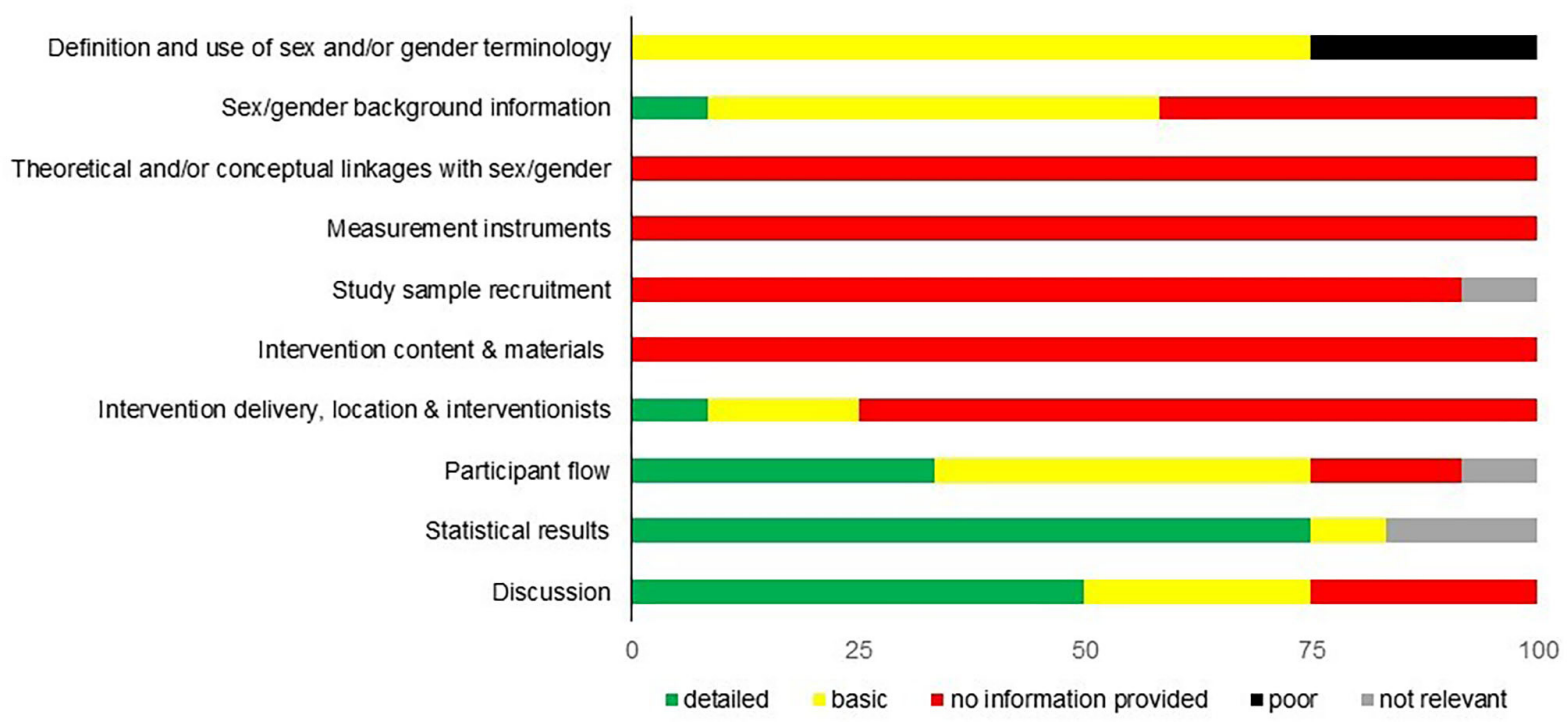
Results of sex/gender checklist.

The results of the sex/gender assessment are presented in Figure 2. In all intervention studies, no information was provided regarding the items *theoretical and/or conceptual linkages with sex/gender, measurement instruments*, and *intervention content and materials*. The item *study sample recruitment* was also rated as “no information provided” in most studies (75%). In single sex/gender studies (Dewar et al., 2014; Dubuy et al., 2014), this item was not relevant. Three studies got a basic or detailed rating for the item *intervention delivery, location*, and *interventionist*. For example, in the DOiT intervention program (Singh et al., 2009; van Nassau et al., 2014), the research team always consisted of two research assistants of whom one performed the anthropometric measurements for female and one for male adolescents. Three studies used sex/gender terminology interchangeably and were rated “poor” in the item *definition of sex/gender* (Cui et al., 2012; Bungum et al., 2014; van Nassau et al., 2014). “Detailed” reporting of sex/gender aspects was mostly realized in the items *statistical results* (75%) and *discussion* (50%). In the discussion, for example, Haerens et al. (2007a) not only reflected information on the identified differences between boys and girls but also stated that “increasing physical activity in girls requires intervention strategies that are tailored for girls.” The rating “basic” was mostly reported in the items *definition and use of sex and/or gender terminology* (75%) and *sex/gender background information regarding the research question* (50%). For example, Dewar et al. (2014) used only the term “gender” (item 1 rated “basic”) and provided information on sex/gender differences in reaching the PA guidelines in the theoretical background (item 2 rated “basic”).

### Intervention Effects in Relation to Considerations of Sex/Gender

We analyzed the relationship between the effects of intervention and sex/gender consideration in view of the results of the sex/gender checklist, which indicated the extent to which studies have taken sex/gender into account. A semiquantitative analysis of intervention effects in relation to the results of sex/gender checklist is presented in Table 3. Sex/gender rating for all included studies is presented for the 10 items of the sex/gender checklist. An average sum rating is provided according to the five possible categories (detailed, basic, no info, poor, and not relevant) to enable a comparison between effective and non-effective intervention programs. If a study is presented more than once, it reflects that different AC outcomes were addressed within the study and that these outcomes showed different intervention effects.

**Table 3:** Intervention effects in relation to considerations of sex/gender in the included studies.

**Author, year of publication**	**Outcome**		**SA**	**Items of sex/gender checklist**										**Sum of ratings**				
				**1**	**2**	**3**	**4**	**5**	**6**	**7**	**8**	**9**	**10**	**Detailed**	**Basic**	**No info**	**Poor**	**Not relevant**
**Studies With Different Intervention Effects in Boys and Girls**																		
Bungum et al. (2014)	ACS	in favor of girls	d											2	0	7	1	0
Villa-Gonzalez et al. (2017)	ACS (bike)	in favor of boys	d											2	2	6	0	0
Average number of ratings (different effects)														2	1	6.5	0.5	0
**Studies With No Significant Intervention Effects in Both Boys and Girls**																		
Cui et al. (2012)	ACS		t											0	4	5	1	0
	PC		t															
Duncan et al. (2011)	ACS		i											1	3	6	0	0
Filho et al. (2016)	ACS		i											3	1	6	0	0
Haerens et al. (2007a)	Leisure time AC		i											3	2	5	0	0
Haerens et al. (2007b)	Leisure time AC		i											1	3	6	0	0
Singh et al. (2009)	ACS		d											1	3	6	0	0
van Nassau et al. (2014)	ACS		d											3	1	5	1	0
Vasickova et al. (2013)	AC in general		d											4	1	6	0	0
Villa-Gonzalez et al. (2017)	ACS (frequency)		d											2	2	6	0	0
	ACS (walk)		d															
	PC (car)		d															
	PC (bus)		d															
Average number of ratings (no effects)														2	2.2	5.6	0.2	0
**Single Sex/Gender Studies With Significant Intervention Effects (in Favor of the Intervention Group)**																		
Dewar et al. (2014)	PC		s											1	2	4	0	3
**Single Sex/Gender Studies Without Significant Intervention Effects**																		
Dubuy et al. (2014)	AC in general		s											2	2	5	0	1
Average number of ratings (overall)														1.9	2	5.6	0.3	0.3
Detailed				0	1	0	0	0	0	1	4	9	6					
Basic				9	6	0	0	0	0	2	5	1	3					
No information provided				0	5	12	12	11	12	9	2	0	3					
Poor				3	0	0	0	0	0	0	0	0	0					
Not relevant				0	0	0	0	1	0	0	1	2	0					

*SA, statistical analysis; s, single sex/gender; d, sex/gender disaggregated; i, interaction; t, tested; AC, active commuting; ACS, active commuting to school; PC, passive commuting; 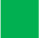, detailed; 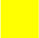, basic; 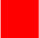, no information provided; 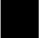, poor; 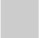, not relevant*.

Overall, three studies demonstrated significant effects on AC outcomes in either boys or girls with one single sex/gender study including only girls (Dewar et al., 2014). One of these studies concluded that overall effects favored girls only (Bungum et al., 2014), while one reported that boys were favored (Villa-Gonzalez et al., 2017). No study reported similar intervention effects in boys and girls. No significant changes in AC were reported in nine intervention studies. Semiquantitative analysis revealed that studies with and without significant intervention effects did not differ with regard to the ratings of the sex/gender checklist. In studies that reported significant effects, statistical analyses of sex/gender were either disaggregated for sex/gender or based on a single sex/gender sample.

## Discussion

This systematic review provides a current overview on the effectiveness of AC intervention studies among children and adolescents aged between 3 and 19 years, which have taken sex/gender into account. To the best of our knowledge, this is the first systematic review that shows how sex/gender has been considered in intervention studies on AC promotion. In total, we identified 12 studies that investigated the promotion of PA in terms of AC and addressed sex/gender aspects at least within their statistical analysis. Within these studies, we examined how sex/gender was considered in background and concepts, study design, intervention planning and delivery, and presentation, as well as interpretation of findings. Overall, the sex/gender checklist showed that the aspects of sex/gender were rarely considered. No differences were found in the results of the sex/gender checklist between studies with and without intervention effects.

### Consideration of Sex/Gender in Intervention Studies on Active Commuting

The majority of the included studies did not adequately reflect sex/gender aspects. Statistical results were rated as at least basic in all intervention studies, as the reporting of sex/gender differences in the results was *a priori* defined inclusion criterion of the current systematic review. Even though the overall rating of sex/gender aspects was insufficient, the included studies provide suggestions to consider these aspects in intervention studies of AC. To address sex/gender aspects in terms of sex/gender background information in detail, authors should provide information about not only existing differences in AC (Haerens et al., 2007a,b; Dubuy et al., 2014) but also the reason for the differences in boys' and girls' behavior, as provided by Vasickova et al. (2013). With regard to intervention planning and delivery, for example, Cui et al. (2012) balanced peer leaders by sex/gender in their peer-led intervention study.

### Effectiveness in Relation to the Consideration of Sex/Gender

Our analysis provided initial information regarding sex/gender in intervention studies on AC; however, it remains elusive as to how these aspects influence the effectiveness of the intervention. As the main aim and focus of this systematic review was on sex/gender aspects in intervention studies on AC rather than summarizing the effects of intervention studies on AC in children and adolescents, conclusions of the overall effectiveness were limited. Many primary studies that address interventions on AC had to be excluded due to no or inadequate consideration of sex/gender in the statistical analysis. Other systematic reviews of AC interventions provide promising information of effectiveness regardless of sex/gender (Chillón et al., 2011; Villa-Gonzalez et al., 2018; Jones et al., 2019), but they fail to report the specific effectiveness of interventions for boys and girls. To overcome the existing gap in AC as well as overall PA between boys and girls worldwide (Guthold et al., 2020), it is important to consider how each intervention affects boys and girls specifically. In their systematic review and meta-analysis, Jones et al. (2019) identified 17 studies that focused on interventions promoting ACS in children; however, only two included studies provided information about sex/gender and compared boys and girls. Most studies adjusted for sex/gender in their statistical analysis or did not address sex/gender aspects at all. Thus, based on previous intervention studies on AC, it remains unclear if girls and boys profit the same way. Most notably, through our systematic review, we identified a current research request for future intervention studies to address sex/gender aspects and to report effects for boys and girls separately.

### Addressing Behavior-Specific and Sex/Gender-Sensitive Determinants to Promote Active Commuting

One factor that may limit the effectiveness of the intervention studies included in this review is that only two included studies had the primary aim to promote AC. All other studies focused on overall PA, occasionally combining it with other health behaviors such as sedentary and dietary behavior. Our results reveal that the two studies, which focused primarily on increasing ACS, resulted in a significant effect on ACS (Bungum et al., 2014; Villa-Gonzalez et al., 2017). All remaining studies that focused on overall PA were not successful in promoting AC, except from one that showed positive effects (Dewar et al., 2014).

As Giles-Corti et al. (2005) proposed, studies on domains of PA should always address behavior-specific predictors. Accordingly, the applied theoretical background, the addressed determinants for behavior change, as well as the suitability for the target group contribute to the effectiveness of an intervention. Specific and detailed knowledge of the determinants regarding the behavior of interest—in our case, AC—is necessary to develop effective intervention programs (Sallis et al., 2000). As the aim of most included studies was to increase overall PA, the intervention programs were based on determinants that possibly influence overall PA but not AC specifically, although AC was assessed as one outcome. In general, a supportive environment in terms of social support from peers and family and built environmental attributes are crucial for supporting AC in both girls and boys (Panter et al., 2010; Ikeda et al., 2018; Aranda-Balboa et al., 2019).

To specify intervention concepts, material, and delivery, not only should the general determinants of the behavior of interest be detected in advance but also differences in determinants in boys and girls. For example, in one included single sex/gender study (Dubuy et al., 2014), the primary study population consisted of boys and girls. However, due to a limited number of girls in the intervention group, girls were excluded from further analyses. The authors argued that the intervention program using professional football players to promote PA is more attractive to boys. To overcome the missing attractiveness of intervention programs either for girls or for boys, future interventions should deeply take into account measures supporting girls and boys.

With regard to active transport, girls are less likely to actively commute to school or to leisure activities than boys (McDonald, 2007; Leslie et al., 2010; Oreskovic et al., 2014). Correspondingly, girls are driven to school more frequently than boys (Colley and Buliung, 2016). This sex/gender gap in active transport has already existed for several years and has increased constantly from 1986 to 2011 (Colley and Buliung, 2016). Additionally, girls accumulate more light PA in active transport, while boys are significantly more active and show more moderate-to-vigorous PA (MVPA) during active transport (Martinez-Martinez et al., 2019; Remmers et al., 2020). Thus, the potential to increase AC and to accumulate more MVPA is especially prominent in girls. For this reason, the knowledge of and the consideration of sex/gender-specific individual, social, and physical environmental predictors is required in the planning, delivery, and implementation of an intervention study on AC (Sirard and Slater, 2008).

As potential starting points of interventions aiming to promote AC in children and adolescents, primary studies identified sex/gender-specific determinants of AC that could be addressed in intervention studies. For example, recreational facilities close to home, distance to school, and a higher perceived safety of the neighborhood predict the level of PA accumulated while traveling actively for both boys and girls (Guliani et al., 2015). However, sex/gender-based differences in determinants of AC have also been identified. For boys, parental perception of automobile safety, heavy traffic near school, missing sidewalks, land use mix, perceived presence of public parks, and social support of friends are correlated with AC (Nelson and Woods, 2010; Guliani et al., 2015). In the included study by Villa-Gonzalez et al. (2017), the applied intervention program, which addressed the aspect of safety, led to an increase in cycling for boys.

In general, girls are less interested toward physical exercise than boys and manifest more barriers such as laziness (Portela-Pino et al., 2019). Thus, determinants of AC in girls are more embedded in the social environment, which motivates them to commute actively. Factors that impact the level of walking in girls include spending time with and talking to friends, accompanying family members, parental restrictions, stranger danger, shops within walking distance, and paths separate from the road (Nelson and Woods, 2010; Kirby and Inchley, 2013). Based on these findings, it becomes obvious that interventions to promote AC in girls and boys should differ in terms of intervention content.

Furthermore, different modes of commuting and travel preferences should be taken into account (McDonald, 2012). Cycling is typically less common in girls than in boys (Leslie et al., 2010; McDonald, 2012). A study with adults revealed that women are more concerned about safety issues, being able to carry daily items, or clothing, such as wearing a helmet and the need to fix their hair upon arrival (Twaddle et al., 2010). Although women or girls prefer walking and are less motivated to use bikes to commute actively, the promotion of cycling in girls is highly demanded as it is more beneficial for health than walking (Shaw et al., 2020). For example, a primary study included in this review revealed that offering the opportunity to ride a bike to school as part of the Nevada Moves Day was successful in increasing the frequency of girls' cycling to school and closing the gap of the prevalence of cycling between boys and girls (Bungum et al., 2014). Additionally, culturally determined preferences in travel behavior need to be addressed in intervention programs. A study from the Netherlands showed that adolescents from non-Western ethnicity walk to school (20%) or are mostly non-active commuters (39%), while native Dutch mostly cycle to school (54%) (Bere et al., 2008).

However, the current evidence on sex/gender-related determinants of AC is limited, as, similarly to intervention studies, sex/gender aspects are not considered in systematic reviews (Pont et al., 2009; Lu et al., 2014; Ikeda et al., 2018; Aranda-Balboa et al., 2019) or are inadequately reported in primary studies. For example, in a systematic review evaluating social and physical determinants of children's independent and active mobility with regard to sex/gender, only seven of the 27 primary studies reported results that were disaggregated for boys and girls (Marzi et al., 2018).

### Relevance for Policymakers and Practitioners

Although conclusions based on the effectiveness of the included studies are limited, this systematic review can support policymakers and practitioners in developing further interventions to promote AC. The existing knowledge on sex/gender-specific determinants needs to be combined with the evidence of successful AC interventions to enable the promotion of AC in girls and boys as well as overcoming the gender gap (Colley and Buliung, 2016). In the development of interventions, boys' and girls' requirements must be addressed. With parental involvement and an improvement of traffic safety and pedestrian infrastructure, AC can be promoted in both girls and boys. A special focus should be placed on the promotion of cycling, as it is less frequent in girls (Simons et al., 2017; Shaw et al., 2020) and, as a mode of commuting, is more effective in stimulating PA than walking (Shaw et al., 2020).

### Strengths and Limitations

This review systematically assessed how sex/gender aspects are considered in interventions that aimed to promote AC/ACS in children and adolescents. No previous review appraised the extent to which the studies have considered sex/gender with a comprehensive checklist, nor has one analyzed the effectiveness in terms of sex/gender. Furthermore, we were able to identify a range of different programs on AC through our search strategy in 11 databases and the assistance of a librarian of the Cochrane Group. Another strength of this systematic review was using the PRISMA statement to improve the reporting quality.

Despite our efforts to gather the best evidence available, this review has some limitations. The review is restricted to English-language articles and peer-reviewed journal articles, and thus, results of other intervention studies published in other languages or literary forms were excluded. Regarding the considerations of sex/gender aspects in the primary studies, we were not able to identify whether these aspects were neglected or simply reported in insufficient detail. These limitations can lead to bias and an undervaluation of sex/gender considerations. To rate and consider sex/gender aspects in intervention studies and to learn from effective interventions on AC, the reporting of sex/gender aspects is a prerequisite. In correspondence to the results of other systematic reviews on interventions on AC (Villa-Gonzalez et al., 2018), our risk of bias assessment rated the majority of included studies as weak. It is also worth mentioning that conclusions should be interpreted carefully, as a meta-analysis was not possible due to a high amount of heterogeneity in primary studies. Finally, we focused only on the binary characterization of gender (boys and girls) because none of the primary studies included gender-diverse participants.

## Conclusion

Until now, no evidence can be provided on the relationship between the effectiveness of intervention studies, which aim to promote AC in children and adolescents, while taking sex/gender into account. The lack of such information can be attributed to the limited number of primary studies that have addressed sex/gender aspects. Similarly, intervention studies have failed to carefully consider these aspects. It has, however, been determined that conditions and barriers of AC differ between boys and girls. Future research should improve reporting and further consider sex/gender in AC research and promote interventions to combat the current sex/gender inequity in AC.

## Data Availability Statement

The original contributions presented in the study are included in the article/Supplementary Material, further inquiries can be directed to the corresponding author/s. Data on the genEffects project as well as the sex/gender checklist are available on request from the project leader, Prof. Dr. Yolanda Demetriou (yolanda.demetriou@tum.de).

## Author Contributions

IM and SE prepared the draft for the manuscript and IM finalized it. SE, CB, and CS performed the searches in consultation with a librarian and extracted the data, conducted the screening of the search, appraised the study quality, and conducted the grading with the sex/gender checklist. AR, JB, and YD supervised this process, secured the funding for the study, and conceived the genEffects project. All authors were involved in the development of the sex/gender checklist and the search strategy, contributed to the interpretation of the results, critically reviewed the manuscript, and approved the final manuscript.

## Conflict of Interest

The authors declare that the research was conducted in the absence of any commercial or financial relationships that could be construed as a potential conflict of interest.

## Abbreviations

AC: active commuting
ACS: active commuting to school
PA: physical activity.
