# Supplementary material for: Interventions Aiming to Promote Active Commuting in Children and Adolescents: An Evaluation From a Sex/Gender Perspective
Source: Front Sports Act Living. 2020 Nov 26;2:590857. doi: 10.3389/fspor.2020.590857 (PMC7739596; doi:10.3389/fspor.2020.590857)
Supplement: Supplementary file 1 [file Table_1.DOCX]

Supplementary Material 1

Additional file 1: Sex/gender checklist including categories, items and their definitions

| **Category** | **Item** | **Definition** |
| --- | --- | --- |
| **Background and concepts** | Definition and use of sex and/or gender terminology | Is the use of sex and/or gender terminology defined in the study? |
|  | Sex/gender background information regarding the research question (e.g. prevalence, strength of association) | Is sex/gender background information regarding the research question taken into account? |
|  | Theoretical and/or conceptual linkages with sex/gender | Is sex/gender linked up with the theory/concept of the intervention? |
| **Study design** | Measurement instruments | Are the measurement instruments valid and reliable for sex/gender groups? |
|  | Study sample recruitment | Is the necessity of sampling for sex/gender taken into account? |
| **Intervention planning and delivery** | Intervention content & materials (e.g. brochures, leaflets, plans of sessions) | Is/are the intervention content/materials inclusive for sex/gender? |
|  | Intervention delivery, location &  interventionists | Is the intervention sex/gender-inclusive regardless the modes of intervention delivery, location and the person carrying out the intervention (instruction/training of implementing persons to be aware of sex/gender-inclusive aspects such as sex/gender-inclusive language)? |
| **Presentation of findings** | Participant flow | Is a participant flow chart provided that takes sex/gender into account according to the CONSORT Statement (eligibility, estimation of sample size (baseline), dropout rates (post-test, follow-up))? |
|  | Statistical results | Are sex/gender differences and/or similarities described regarding the outcomes? |
| **Interpretation of findings** | Discussion | Are the findings reflected with respect to sex/gender? |
