# Supplementary material for: Interventions Aiming to Promote Active Commuting in Children and Adolescents: An Evaluation From a Sex/Gender Perspective
Source: Front Sports Act Living. 2020 Nov 26;2:590857. doi: 10.3389/fspor.2020.590857 (PMC7739596; doi:10.3389/fspor.2020.590857)
Supplement: Supplementary file 2 [file Table_2.DOCX]

Supplementary Material 2

Additional File 2: Data extraction of included studies

| **Author, year of publication**  **Country** | **Sample size (n)**  **Age (M±SD; years)**  **Sex/gender (%male)**  **Setting** | **study design**  **duration of intervention**  **analysis of sex/gender** | **Intervention description** | **AC outcome**  **definition**  **measure** | **Results for AC** |
| --- | --- | --- | --- | --- | --- |
| Bungum et al. (2014)  USA | nI=638; nC=698  age=5-11y  sex/gender unknown  school | CT/ Quasi experiment  Short term (1 week)  disaggregated | **Intervention:**  School participated in NMD (Nevada Moves Day) to encourage active travel to school  media publicity to reach students  6 strategies delivered by physical education teacher to promote active travel in 3 days as a part of regular announcements  **Control:** no intervention | AC to school  Objective: oberservation and counting of active commuters | **Intervention effects between groups**  ACS rates were higher for Intervention group at NMD day (Chi²=27.2; p<0.001)  Intervention Group: 17.9% ACS  Control Group: 7.0% ACS  ACS rates returned to baseline level in both groups (Chi²=0.66; p=0.41) at post intervention  **Within groups**  Significant increase in bicyclists (Chi²=11.92; p<0.001) and other wheels (Chi²=10.98; p=0.02) in the intervention school from Baseline to post intervention  **Sex/gender**  Number of girls who used ACS doubled from baseline to NMD (Chi²=13.5; p<0.05)  Number of boys using ACS did not significantly increase (Chi²=1.79;p=0.056) |
| Cui et al. (2012)  China | nI=346; nC=336  age=12.6±0.5y  51.75% male  school | Cluster-RCT  Short term (4 weeks)  tested | **Intervention:**  Four-component peer-led health programme (food choice, PA & SB, carbonated drinks and goal setting)  Learning activities were designed to be conducted in a variety of ways: presentations, video watching, group discussion, games, experiments, lifestyle practice, skit playing, quiz show  Peer leader’s manual to describe the structural activities  **Control:** no intervention | AC to and from school  AC in general (inactive travel)  Subjective: 7-day physical activity questionnaire | **Intervention effects between groups**  No significant differences in ACS and passive commuting between control and intervention group after 3 months (p=0.82; p=0.22) and 7 months (p=0.07;p=0.32))  **Sex/gender**  no significant effects |
| Dewar et al. (2014)  Australia | nI=179; nC=179  age=13.2± 0.5y  only girls  school | Cluster-RCT  Moderate term (12 months)  single sex/gender | **Intervention:**  School sport sessions, interactive seminars, nutrition workshops, lunch-time PA, PA and nutrition handbooks, parent newsletters, pedometers for self-monitoring, text messaging for social support  **Control:** no intervention | AC in general (inactive travel)  Subjective: Adolescent Sedentary Activity Questionnaire | **Intervention effects between groups**  change in time for inactive travel was in favor of the intervention group with a between group difference of −8.6 min/day (95%CI:−18.1-1.0, p=0.07) |
| Dubuy et al. (2014)  Belgium | nI=146; nC=268  age(I)=12.6±1.0y; age(C)=12.1±1.5y  only boys  professional football clubs and schools | CT  Moderate term (4 months)  single sex/gender (boys) | **Intervention:**  Start and end clinics at football clubs  4-month-school-based program: school and classroom activities (e.g. active playgrounds and activity breaks during lessons)  2 main topics: healthy diet and PA  2 letters from professional football players reminding the pupils of the importance of regular PA  **Control:** no intervention | AC to and fom school  Subjective: Flemish Physical Activity Questionnaire | **Intervention effects between groups**  no significant time by condition interaction effect was found for ACS (F=0.16; p=0.70) |
| Duncan et al. (2011)  Australia | nI=57; nC= 40  age=9-11y  36.82% male  school | Cluster-RCT  Short term (6 weeks)  interaction | **Intervention:**  Each child received homework booklet organized into 5 PA topics  Tasks were designed to encourage parental participation and family involvement  3 homework options for PA; children were required to complete at least one task per week for PA/week (e.g., walking from and to school)  password-protected healthy homework-website  In-class exercises for three 1.5 hours session each week  (including one session reviewing the previous week's homework)  **Control:** no intervention | AC to and from school  Subjective: diary | **Intervention effects between groups**  No significant differences in ACS between intervention and control group at Baseline and Follow-up  **Within group**  Decrease in active transport time on weekdays (p=0.028) in control group  **Sex/gender**  no significant effects |
| Filho et al. (2016) | nI=548; nC=537  age=11-18y  51.5% male  school | Cluster-RCT  Moderate term (4 months)  interaction | **Intervention:**  Training and activities in general curriculum  PE teacher-specific training (manual with lesson plans and handouts; production of posters and text by adolescents with health issues content)  Opportunities in the school environment to engage in PA  Health education in school community (e.g. posters)  **Control:** no intervention | AC to school  Subjective: questionnaire | **Intervention effects between groups**  No significant intervention effects on ACS (adj OR=1.3, 95%CI: 0.53-3.19, p=0.48)  **Sex/gender**  no significant effects |
| Haerens et al. (2007)  Belgium | nI+Parental support=1194; nI=911; nC=735  age=13.1±0.8y  62% male  school | Cluster-RCT  Moderate term (9 months)  interaction | **Intervention:**  More opportunities to be physically active during school breaks, at noon or after-school hours  Extra PA on after-school hours and at noon  Extra sport materials  Personal PA advice  Parents interactive group meeting on PA and relationship with health  **Control:** no intervention | leisure time AC  Subjective: Flemisch Physical Activity Questionnaire | **Intervention effects between groups**  No significant intervention effects on leisure time AC  **Sex/gender**  A significant gender-by-condition interaction was found (F=3.9, p≤.05)  boys: n.s. (F<1.2)  girls in control group: decrease in AC on average 4 minutes daily (F=12.1, p<0.001, d=0.28) |
| Haerens et al. (2007)  Belgium | nI=139; nC=142  age(I)=13.2±0.8y age(C)=13.2±0.5y  52% male  school | Cluster-RCT  Short term (3 months)  interaction | **Intervention:**  Computer-tailored intervention (provided on CD´s) with personal PA advice to encourage students to increase PA (for students who did not meet PA guidelines) or keep up with their PA habits  **Control:** no intervention | leisure time AC  Subjective: Flemisch Physical Activity Questionnaire | **Intervention effects between groups**  No significant intervention effects on leisure time AC (F≤2.5)  **Sex/gender**  No significant effects |
| Singh et al. (2009)  Netherlands | nI=623; nC=476  age(I_girls)=12.6±0.5y  age(I_boys)=12.8±0.5y age(C_girls)=12.7±0.5y age(C_boys)=12.9±0.5y  46.7% male (I)  53% male (C)  school | Cluster-RCT  Moderate term (8 months)  disaggregated | **Intervention:**  consisted of an individual component (i.e., an educational program covering 11 lessons for the courses of biology and physical education) and an environmental component (i.e., encouraging schools to offer additional physical education classes and advice for schools on changes in and around school cafeterias).  DOit-Program: Intervention Mapping protocol, which facilitates a systematic process of designing health promotion interventions and is based on theory and empirical evidence  **Control:** no intervention | AC to school  Subjective: questionnnaire | **Intervention effects between groups**  No significant intervention effect on ACS in girls and boys  **Sex/gender**  No significant effects |
| van Nassau et al. (2014)  Netherlands | nI=1002; nC=484  age=12-14y  sex/gender unknown  school | Cluster-CT  long term (20 months)  disaggregated | **Intervention:**  DOiT-Program: school-based obesity prevention programme for 12 to 14-year olds, developed according to the Intervention Mapping protocol  focused on five EBRBs: reducing intake of sugar containing beverages; reducing intake of high-energy snacks/sweets; reducing screen time; increasing levels of physical activity (i.e., active transport to school and sports participation) and consuming a daily breakfast  **Control:** no intervention | AC to school  Subjective: questionnaire | **Intervention effects between groups**  No significant intervention effect on ACS  **Sex/gender**  No significant effects |
| Vasickova et al. (2013)  Poland | nI=258; nC=237  age(I_girls)=15.7±0.8y age(I_boys)=15.6±0.8y age(C_girls)=15.9±0.9y age(C_boys)=15.7±0.8y  44% male  school | Cluster-RCT  short term (4 weeks)  disaggregated | **Intervention:**  4-week, self-monitoring, physical activity and educative programme  Motivational recording brochures (record tables for data from pedometer, possibility of graphic expressions of daily steps, record tables for movement / inactivity)  **Control:** no intervention | AC in general  Subjective: Polish Version of the International Physical Activity Questionnaire | **Intervention effects between groups**  not reported  **Sex/gender**  No significant differences between boys and girls in ACS before and after the intervention |
| Villa-Gonzalez et al. (2017)  Spain | nI=141; nC=110  age=9.1y  49% male | CT/ Quasi experiment  Moderate term (6 months)  disaggregated | **Intervention:**  focused on increasing the frequency of ACS  teachers and the research team implemented the intervention program at each school  all activities were carried out in the classroom and in the school neighborhood  **Control:** no intervention | AC to and from school  Subjective: self-report questionnaire | **Intervention effects between groups**  No significant interaction between assessment point and frequency of AC and modes of commuting were found  **Sex/gender**  Boys in the IG had higher rates of cycling to school than boys of the CG (p = 0.04) at follw-up  Girls in the IG avoided increase in the rates of passive commuting at follow up, which increased in the CG among girls for car (p<0.05) and bus use (p<0.05) |

Note: nI = sample size intervention group; nC = sample size control group; y = years; AC = active commuting; ACS = active commuting to school; CT = controlled trial, RCT = randomized controlled trial

References

Bungum, T.J., Clark, S., and Aguilar, B. (2014). The Effect of an Active Transport to School Intervention at a Suburban Elementary School. *American Journal of Health Education* 45(4)**,** 205-209. doi: 10.1080/19325037.2014.916635.

Cui, Z., Shah, S., Yan, L., Pan, Y., Gao, A., Shi, X., et al. (2012). Effect of a school-based peer education intervention on physical activity and sedentary behaviour in Chinese adolescents: a pilot study. *BMJ Open* 2(3). doi: 10.1136/bmjopen-2011-000721.

Dewar, D.L., Morgan, P.J., Plotnikoff, R.C., Okely, A.D., Batterham, M., and Lubans, D.R. (2014). Exploring changes in physical activity, sedentary behaviors and hypothesized mediators in the NEAT girls group randomized controlled trial. *J Sci Med Sport* 17(1)**,** 39-46. doi: 10.1016/j.jsams.2013.02.003.

Dubuy, V., De Cocker, K., De Bourdeaudhuij, I., Maes, L., Seghers, J., Lefevre, J., et al. (2014). Evaluation of a real world intervention using professional football players to promote a healthy diet and physical activity in children and adolescents from a lower socio-economic background: a controlled pretest-posttest design. *BMC Public Health* 14**,** 457. doi: 10.1186/1471-2458-14-457.

Duncan, S., McPhee, J., Schluter, P., Zinn, C., Smith, R., and Schofield, G. (2011). Efficacy of a compulsory homework programme for increasing physical activity and healthy eating in children: the healthy homework pilot study. *The international journal of behavioral nutrition and physical activity* 8**,** 127. doi: 10.1186/1479-5868-8-127.

Filho, V.C., da Silva, K.S., Mota, J., Beck, C., and da Silva Lopes, A. (2016). A Physical Activity Intervention for Brazilian Students From Low Human Development Index Areas: A Cluster-Randomized Controlled Trial. *J Phys Act Health* 13(11)**,** 1174-1182. doi: 10.1123/jpah.2016-0113.

Haerens, L., De Bourdeaudhuij, I., Maes, L., Cardon, G., and Deforche, B. (2007). School-based randomized controlled trial of a physical activity intervention among adolescents. *J Adolesc Health* 40(3)**,** 258-265. doi: 10.1016/j.jadohealth.2006.09.028.

Singh, A.S., Chin, A.P.M.J., Brug, J., and van Mechelen, W. (2009). Dutch obesity intervention in teenagers: effectiveness of a school-based program on body composition and behavior. *Arch Pediatr Adolesc Med* 163(4)**,** 309-317. doi: 10.1001/archpediatrics.2009.2.

van Nassau, F., Singh, A., Cerin, E., Salmon, J., Mechelen, W., Brug, J., et al. (2014). The Dutch Obesity Intervention in Teenagers (DOiT) cluster controlled implementation trial: Intervention effects and mediators and moderators of adiposity and energy balance-related behaviours. *International Journal of Behavioral Nutrition and Physical Activity* 11. doi: 10.1186/s12966-014-0158-0.

Vasickova, J., Groffik, D., Fromel, K., Chmelik, F., and Wasowicz, W. (2013). Determining gender differences in adolescent physical activity levels using IPAQ long form and pedometers. *Ann Agric Environ Med* 20(4)**,** 749-755.

Villa-Gonzalez, E., Ruiz, J.R., Mendoza, J.A., and Chillon, P. (2017). Effects of a school-based intervention on active commuting to school and health-related fitness. *BMC Public Health* 17(1)**,** 20. doi: 10.1186/s12889-016-3934-8.
